# An Integrated Profiling of Liver Metabolome and Transcriptome of Pigs Fed Diets with Different Starch Sources

**DOI:** 10.3390/ani14223192

**Published:** 2024-11-07

**Authors:** Miao Yu, Zhenming Li, Yiyan Cui, Ting Rong, Zhimei Tian, Dun Deng, Zhichang Liu, Ruiyang Zhang, Xianyong Ma

**Affiliations:** 1 Institute of Animal Science, Guangdong Academy of Agricultural Sciences, State Key Laboratory of Swine and Poultry Breeding Industry, Key Laboratory of Animal Nutrition and Feed Science in South China, Ministry of Agriculture, Guangdong Public Laboratory of Animal Breeding and Nutrition, Guangdong En-gineering Technology Research Center of animal Meat quality and Safety Control and Evaluation, Guangzhou 510640, China; yumiao@gdaas.cn (M.Y.); lizhenming@gdaas.cn (Z.L.); cuiyiyan@gdaas.cn (Y.C.); rongting@gdaas.cn (T.R.); tianzhimei@gdaas.cn (Z.T.); dengdun@gdaas.cn (D.D.); liuzhichang@gdaas.cn (Z.L.); 2 College of Animal Science and Veterinary Medicine, Shenyang Agricultural University, Shenyang 110866, China

**Keywords:** liver, metagenomics, metabolome, starch sources, pigs

## Abstract

Diets containing higher-amylose-content starches were proved to have some beneficial effects on monogastric animals, such as promoting the proliferation of intestinal probiotics. However, current research on the effects of diets with different starch sources on animals at the extraintestinal level is still very limited. We hypothesized that diets with different starch sources may affect lipid-related gene expression and metabolism in the liver of pigs. This study aimed to use adult pig models to evaluate the effects of diets with different starch sources (tapioca starch, TS; pea starch, PS) on the liver gene expressions and metabolism. In total, 48 growing pigs were randomly assigned to the TS and PS diets with 8 replicate pens/group and 3 pigs per pen. On day 44 of the experiment, liver samples were collected for metabolome and transcriptome analysis. Metabolome data suggested that different starch sources affected (*p* < 0.05) the metabolic patterns of liver. Compared with the TS diet, the PS diet increased (*p* < 0.05) some unsaturated fatty acids and several amino acids or peptide levels in the liver of pigs. Moreover, transcriptome data indicated the PS diets elevated (*p* < 0.05) fatty acid β-oxidation-related gene expression in the liver of pigs, and reduced (*p* < 0.05) unsaturated fatty acid metabolism-related gene expression. The results of quantitative real-time PCR confirmed that the PS diet upregulated (*p* < 0.05) the expression of acyl-CoA dehydrogenase very long chain (ACADVL), carnitine palmitoyl transferase (CPT) 1A, and malonyl-CoA decarboxylase (MLYCD), and downregulated (*p* < 0.05) the expression level of cytochrome P450 2U1 (CYP2U1) and aldehyde dehydrogenase 1B1 (ALDH1B1) in the liver. In addition, the results of a Mantel test indicated the muscle fatty acids were significantly closely correlated (*p* < 0.05) with liver gene expressions and metabolites. In summary, these findings suggest that diets containing higher amylose starches improved the lipid degradation and the unsaturated fatty acid levels in pig livers, and thus can generate some potential beneficial effects (such as anti-inflammatory and antioxidant) on pig health.

## Simple Summary

Starches with different structures had different effects on pig health and metabolism. The present study integrated transcriptome and metabolome analysis to reveal the differences in gene expressions and metabolism in the liver of pigs fed diets with different starch sources (different amylose/amylopectin ratio). The results showed that diets with a higher amylose/amylopectin ratio could increase the degradation of fatty acids and the accumulation of unsaturated fatty acids in the liver.

## 1. Introduction

Starch, the major carbohydrate composition and energy source in the daily diet of human and monogastric animals, is composed of amylose and amylopectin, having different physiological functions [1]. Amylose is a linear polysaccharide formed by glucose units through α-1,4 glycosidic bonds, and the structure of amylopectin contains branched polymer chains of glucose units connected by α-1,6 glycosidic bonds [2,3]. Due to this difference in molecular structure, amylose is not easily digested in the small intestine, thus increasing the starch content that is present in the posterior intestine of monogastric mammals [2,3]. Diets with different starch sources always result in differences in amylose content. For example, compared with maize starch and tapioca starch, pea starch contains a higher amylose content, which makes it somewhat resistant to amylase digestion [4]. Numerous studies confirmed that diets with higher-amylose-content starches had some beneficial effects on human and monogastric animals, such as improving meat quality and promoting the proliferation of intestinal probiotics [5,6,7,8]. Fermentation of high-amylose starch in the posterior intestine leads to an increase in the content of volatile fatty acids that can be utilized by the host, and can more smoothly increase glucose in blood circulation, which is a possible reason why high-amylose starch exerts the above-mentioned beneficial effects [4]. However, current research on the effects of diets with different starch sources on monogastric animals at the extraintestinal level is still very limited.

The liver is considered to play an vital role in the metabolism of three major nutrients (carbohydrates, proteins, and lipids), and is also an important immune organ in mammals [9,10]. Hence, the health status of the liver is closely related to the host. A previous study reported that diets containing higher-amylose-content starches could cause the downregulation of genes related to gluconeogenesis and fat accumulation, while causing the upregulation of genes related to protein deposition using real-time PCR [11]. Another study demonstrated that diets containing higher-amylose-content starches decreased the total lipid and cholesterol level in the liver of growing–finishing pigs [12]. However, these studies mainly centered on the changes in several genes or lipid-related metabolites in the liver, thus resulting in very limited data on the global impacts of dietary starch sources on liver gene expression and metabolism.

Our previous studies showed that diets containing higher-amylose-content starches altered the expression of genes related to lipid metabolism and fatty acid content in muscles of pigs [7]. On this basis, in the present study, we hypothesized that diets with different starch sources may affect lipid-related gene expression and metabolism in the liver of pigs. Therefore, the present study integrated transcriptome and metabolome analysis to reveal the differences in gene expressions and metabolism in the liver of pigs fed diets with different starch sources.

## 2. Materials and Methods

### 2.1. Animal Experiment, Management, and Diets

The present study was a portion of systematic experiments evaluating the impacts of different dietary starches on intestinal microecological environments in pigs, and the specific experiment procedures and designs were described in our published study [6]. Generally, a total of 48 growing barrows (Duroc × Landrace × Large White) were assigned to 2 treatments with 8 replicates per treatment and 3 pigs per replicate. At the start of the experiment, the body weight (BW) of all pigs was 77.0 ± 0.52 kg, and there was no significant difference between the two groups. Tapioca and pea starches were used as the dietary starch sources in two diets in the present study, respectively. Only the starch sources were different, and the other feed ingredients in the two diets were the same. The amylose/amylopectin ratios of the TS and PS diets were 0.11 and 0.44, respectively. The experimental diets were formulated according to the National Research Council (NRC 2012), and the detailed feed composition and nutritional levels are listed in Table 1. The animal experiment lasted for 40 days, and animals were free to access experimental feed and water.

### 2.2. Sample Collection

In the end, after fasting for approximately 12 h, 16 barrow experimental animals (8 piglets/group, 1/pen) were euthanized by electrical stunning, and slaughtered. The liver tissue (approximately 2 g) was cut into pieces using scissors, mix thoroughly, and then sampled. These samples were fast frozen in liquid nitrogen and then stored at −80 °C until further analysis. To balance the cost of the experiment and the number of replicates necessary, 7 liver tissue samples from each group were randomly selected for further transcriptomic and metabolomic analysis.

### 2.3. Metabolome Approach and Data Analysis

In the present study, we used a liquid chromatography–mass spectrometry (LC-MS)-based metabolome approach to determine the liver metabolites in pigs. Liver samples were freeze-dried for 12 h. For preprocessing, freeze-dried samples were thawed out on the ice, and 50 mg samples were mixed with methanol (800 μL) and DL-o-Chlorophenylalanine (10 μL). DL-o-Chlorophenylalanine was used as the internal standard for the correction of peak data. After the mixture was ultrasonicated (40 KHz, 30 min), the samples were centrifuged (12,000× *g*, 4 °C, 15 min) and then the supernatant (200 μL) was placed into a vial for subsequent analysis. Metabolomic detection was performed on an LC-MS system (Thermo, Ultimate 3000LC, Q Exactive, Waltham, MA, USA) equipped with a Hyper gold C_18_ column (100 × 2.1 mm, 1.9 μm). The instrument was used in accordance with the manufacturer’s standard procedures. After the determination, the preprocessed and sorted data included retention time, molecular weight, and peak intensity.

### 2.4. RNA Isolation, Sequencing and Data Analysis

Total RNA was isolated from 100 to 200 mg of frozen liver samples, and tissue samples were homogenized in 1 mL of TRIzol reagent (Takara Bio, Otsu, Japan), as described in a previous study [13]. The reagents and consumables used during the isolation process were RNA-free. After isolation, the concentration, quality, and integrity of the RNA samples were evaluated by a Nanodrop-1000 spectrophotometer (Thermo Fisher Scientific Inc., Waltham, MA, USA) and Agilent 2100 Bioanalyzer (Agilent Technologies, Santa Clara, CA, USA). Only the high-quality RNA samples were subjected for transcriptome sequencing. The sequencing was completed on an Illumina Hiseq 2000 platform and paired-end sequencing was performed.

After the quality filtering of the raw data, sequences were compared to the *Sus scrofa* reference genome using HISAT2 (http://ccb.jhu.edu/software/hisat2/index.shtml (accessed on 4 April 2024)). To construct the trait-related gene co-expression modules, the Weighted Gene Co-expression Network Analysis (WGCNA) tool in the R (v 4.0.4) package was adopted [14]. The enrichment of Gene Ontology (GO) was conducted and the Kyoto Encyclopedia of Genes and Genomes (KEGG) employed, utilizing DAVID 6.8 [15].

### 2.5. cDNA Synthesis, Primers, and Quantitative Real-Time PCR (qRT-PCR)

After RNA extraction, total RNA was reverse-transcribed using a PrimeScript^®^ RT reagent kit with gDNA Eraser (Takara Bio, Otsu, Japan). Triplicate PCR reactions of all determined genes were conducted on a CFX96 Real-time PCR Detection System (Bio-Rad Laboratories, Hercules, CA, USA). The gene expression of each target gene was normalized by the housekeeping gene (β-actin), and then was calculated using the 2^−(ΔΔCt)^ method [16]. The primers for all the genes determined in the present study are listed in Table S1.

### 2.6. Statistical Analysis

All experimental data were initially organized in Excel 2010, and then SPSS software (SPSS v. 20.0, Chicago, IL, United States) was utilized for statistical analysis. Multivariate analysis of the metabolomics data was performed using SIMCA-P + 14.1 software (Umetrics, Umea, Sweden). In particular, the differences in the liver metabolites between the TS and PS diets were analyzed using the partial least squares discriminant analysis (PLS-DA) and orthogonal partial least-squared discriminant analysis (OPLS-DA). The differential metabolites between the TS and PS groups were evaluated through a Variable importance in Projection (VIP, harvested from the PLS-DA analysis) > 1 and *p* < 0.05 (obtained from a Wilcoxon–Mann–Whitney U test).

The differences in gene expressions based on transcriptome data between the TS and PS groups were evaluated using DESeq2 v1.30.1 [17]. After the analysis, we defined differentially expressed genes (DEGs) as genes with an absolute value of fold change > 1 and a false discovery rate (FDR) < 1. The differences in gene expressions conducted using real-time PCR were evaluated using an independent-sample t test. The Mantel test was conducted to test the correlations between liver metabolites and gene expressions and muscle fatty acids. Significant difference was declared when *p* < 0.05.

## 3. Results

### 3.1. Variations in Liver Metabolites in Pigs After Different Dietary Treatments

**Figure 1:**
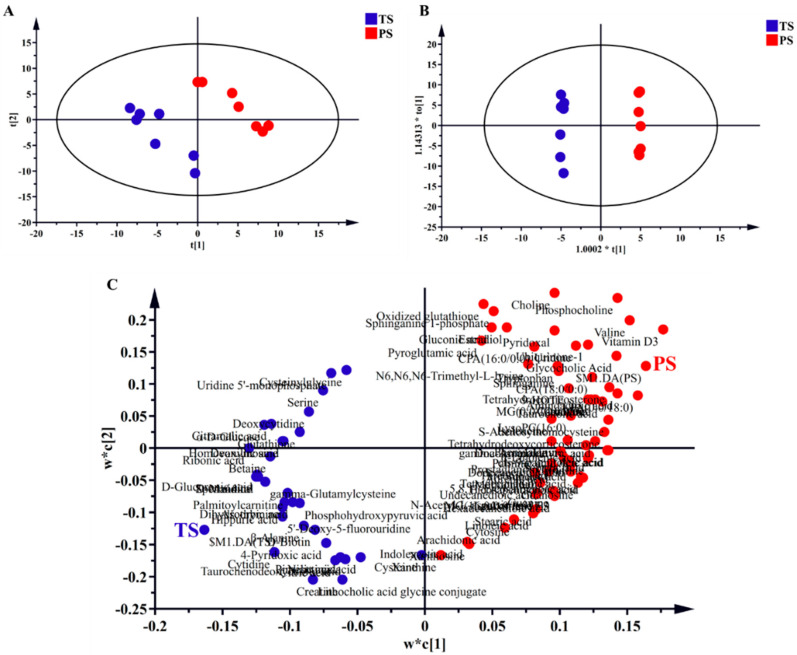
Partial least squares discriminant analysis (PLS-DA; (**A**,**C**) and orthogonal partial least-squares discriminant (OPLS-DA; (**B**)) analysis of liver metabolites. t[1] and t[2] were the first and second principal components of PLS-DA, respectively. 1.0002*t[1] and 1.14313*t_0_[1] were the first and second principal components of OPLS-DA, respectively. w*c[1] and w*c[2] were the first and second principal components of PLS-DA loading plots, respectively. TS, tapioca starch; PS, pea starch.

Through the metabolomic analysis, a total of 198 effective metabolites were detected in the livers of finishing pigs. Next, we conducted PLS-DA and OPLS-DA to estimate the overall impacts of the PS diets. The results of both PLS-DA and OPLS-DA (Figure 1A,B) indicated that the liver samples of different dietary treatments showed clear clusters and separations. The PLS-DA loading plots (Figure 1C) show the distribution condition of liver metabolites with VIP > 1, indicating that closely related liver metabolites differed greatly under different diets. Briefly, the TS diets were closely related with liver metabolites such as cytidine, 5′-Deoxy-5-fluorouridine, and hippuric acid, while the PS diet was closely related with liver metabolites such as vitamin D3, valine, and LPA (0:0/18:0).

**Figure 2:**
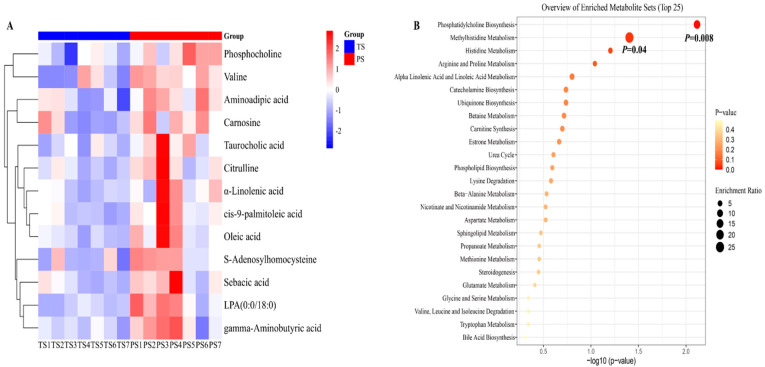
The differential liver metabolites and their enrichment analysis. (**A**) The differential liver metabolites identified from the tapioca starch (TS) and pea starch (PS) groups. Only the metabolites that met the criteria of VIP > 1 and *p* < 0.05 were defined as differential metabolites. (**B**) The enrichment analysis of differential liver metabolites.

In the present study, the liver metabolites that met the criteria of VIP > 1 (harvested from the PLS-DA) and *p* < 0.05 (harvested from statically analysis) were defined as differential metabolites. After statistical analysis, 16 liver metabolites were defined as differential metabolites (Figure 2A). In general, the PS group elevated the concentrations of cis-9-palmitoleic acid, oleic acid, tetrahydrocorticosterone, phosphocholine, S-adenosylhomocysteine, aminoadipic acid, citrulline, LPA (0:0/18:0), carnosine, CPA (18:0/0:0), γ-aminobutyric acid, valine, MG (0:0/14:0/0:0), sebacic acid, α-linolenic acid, and taurocholic acid in the liver.

Next, we conducted the enrichment analysis of differential liver metabolites identified between the two dietary treatments. The results indicated that the pathways including phosphatidylcholine biosynthesis and methylhistidine metabolism were significantly enriched in the present study, and the compounds involved in the above two pathways were mainly phosphocholine and S-adenosylhomocysteine (Figure 2B).

### 3.2. Gene Co-Expression Network of Host Transcriptome, Diets, and Liver Metabolites

**Figure 3:**
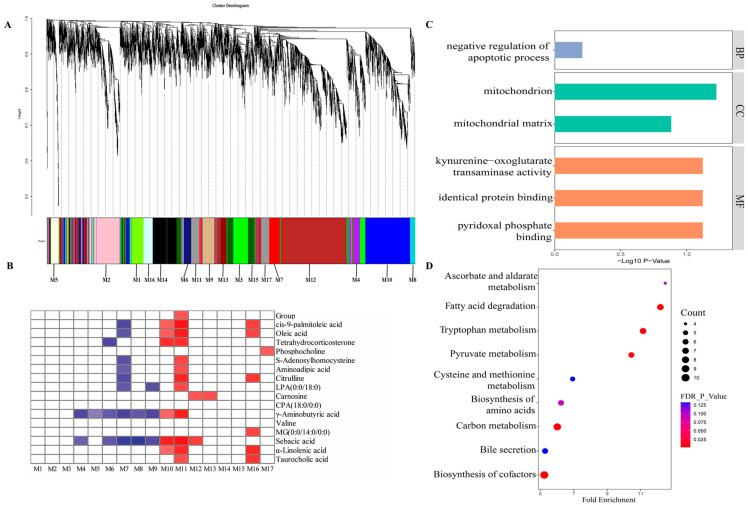
Gene co-expression networks analysis of the correlation of the host transcriptome with host phenotypic traits. (**A**) A dendrogram from the gene co-expression network analysis of liver samples from the TS and PS groups. Modules of co-expression genes were assigned to a color and number (M1 to M17). (**B**) The relationship between dietary groups, liver metabolites, and the 17 gene modules. The cells filled with red indicate a positive correlation, and blue indicate a negative correlation. The darker the color, the greater the correlation coefficient. (**C**) The significantly enriched Gene Ontology category in the M11 module. These three colors in the figure corresponded to BP, CC, and MF, respectively. BP, biological process; CC, cellular component; MF, molecular function. (**D**) The significantly enriched KEGG pathways in the M11 module. TS, tapioca starch; PS, pea starch.

Through the transcriptome sequencing of liver samples, we harvested a total of 653,308,620 high-quality sequences, with an average of 46,664,901 sequences per sample. For a comprehensive understanding of liver transcriptome profiles under the diets with different starch sources, WGCNA was conducted to assess the co-expressed gene modules that were closely related with the host phenotypic traits. The WGCNA suggested the host genes could be divided into 17 gene modules (M1 to M17) (Figure 3A).

### 3.3. Functional Profiles of Host Genes in the M11 Module

Among the identified 17 gene modules, the M11 module contained 228 genes, accounting for 2.13% of total host genes. Our results showed that the M11 module presented the strongest correlations, and was the only gene module positively correlated with the different dietary treatments (Figure 3B). Simultaneously, the host genes in the M11 module were also positively correlated with 11 liver metabolites (cis-9-palmitoleic acid, oleic acid, tetrahydrocorticosterone, S-Adenosylhomocysteine, aminoadipic acid, citrulline, LPA (0:0/18:0), γ-aminobutyric acid, sebacic acid, α-linolenic acid, and taurocholic acid).

Next, we conducted the GO and KEGG enrichment analysis of these host genes in the M11 module. These host genes were apparently enriched in six GO terms (Figure 3C), including negative regulation of pyridoxal phosphate binding, apoptotic process, mitochondrion, mitochondrial matrix, kynurenine–oxoglutarate transaminase activity, and identical protein binding. Moreover, the results of KEGG enrichment analysis (Figure 3D) showed that nine pathways, including tryptophan metabolism, fatty acid degradation, carbon metabolism, pyruvate metabolism, biosynthesis of amino acids, biosynthesis of cofactors, and bile secretion, were significantly enriched.

### 3.4. Expression Pattern of M11 Hub Genes Between Two Dietary Treatments

**Figure 4:**
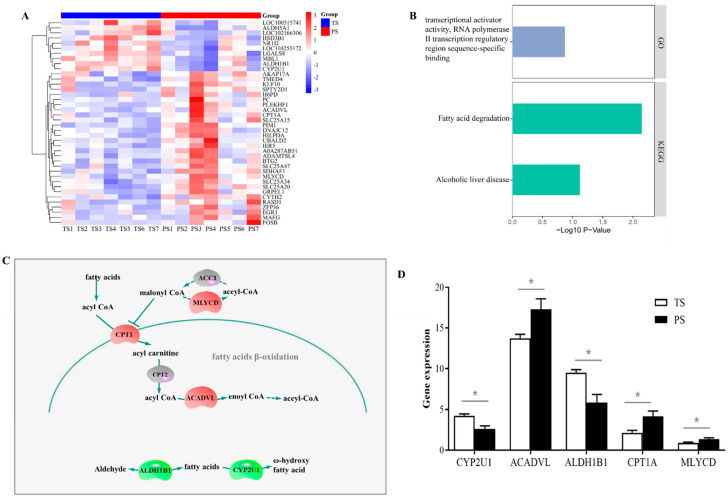
The differentially expressed genes (DEGs) and their functions identified in liver of the TS and PS groups. (**A**) DEGs in the M11 module. (**B**) The significantly enriched Gene Ontology category and KEGG pathways of DEGs. (**C**) The affected DEGs in the pathway of fatty acid degradation. (**D**) The expression of DEGs identified through transcriptome involved in the fatty acid degradation. TS, tapioca starch; PS, pea starch. * *p* < 0.05.

As shown in Figure 4A, 46 genes were considered DEGs between the two dietary treatments in total, consisting of 33 upregulated and 13 downregulated genes with the PS diets. The GO and KEGG pathway enrichment analysis (Figure 4B) suggested these DEGs were significantly enriched in the transcriptional activator activity, RNA polymerase II transcription regulatory region sequence-specific binding (GO term), fatty acid degradation (KEGG pathway), and alcoholic liver disease (KEGG pathway). We summarize the expression of these DEGs involved in the above two KEGG pathways (fatty acid degradation, and alcoholic liver disease) in Figure 4C. Our findings (Figure 4D) indicate that the PS group elevated (*p* < 0.05) the expression of acyl-CoA dehydrogenase very long chain (ACADVL), carnitine palmitoyl transferase (CPT) 1A, and malonyl-CoA decarboxylase (MLYCD), and reduced (*p* < 0.05) the expression level of cytochrome P450 2U1 (CYP2U1) and aldehyde dehydrogenase 1B1 (ALDH1B1) in the liver.

### 3.5. Validation of the Expressions of DEGs Involved in the Fatty Acid Degradation Using the Real-Time PCR Method

**Figure 5:**
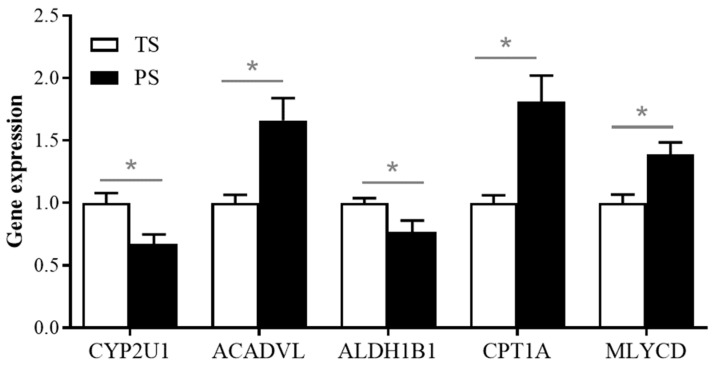
Validation of the expressions of DEGs involved in the fatty acid degradation using the real-time PCR method.* *p* < 0.05.

To validate the reliability of transcriptomic data, we determined the relative expression of DEGs involved in the fatty acid degradation using the real-time PCR method. Our results showed that, consistent with our transcriptomic data, the PS group elevated the expression of ACADVL, CPT1A, and MLYCD (*p* < 0.05), and reduced the expression level of CYP2U1 and ALDH1B1 in the liver (*p* < 0.05) (Figure 5).

### 3.6. The Relationship Between Liver Status (Gene Expression and Metabolites) and Muscle Fatty Acids in Pigs

**Figure 6:**
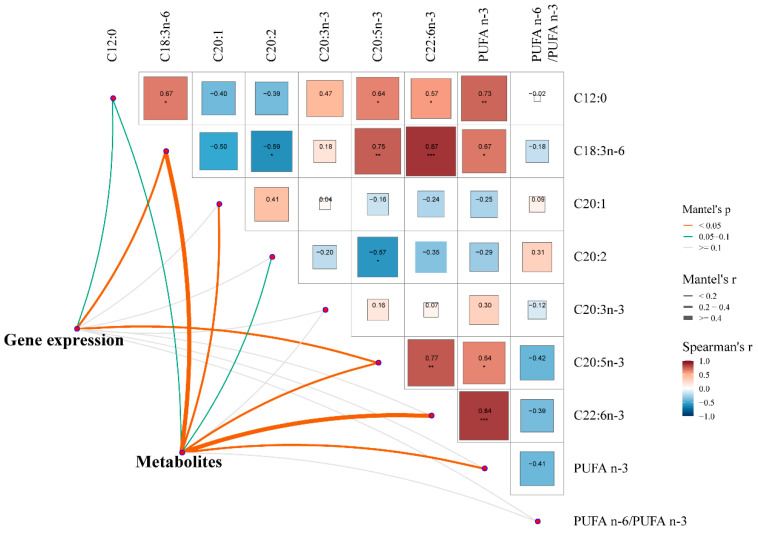
Mantel test for correlation between liver status (gene expression and metabolites) and muscle fatty acids in pigs. Edge width represents correlation r value obtained by Mantel test, and edge color corresponds to statistical P value based on 9999 permutations. * *p* < 0.05; ** *p* < 0.01; *** *p* < 0.001.

The fatty acid profiles in the longissimus thoracis muscle were described in our previous study [7]. Briefly, the PS group increased (*p* < 0.05) the concentrations of C12:0, C18:3n-6, C20:3n-3, C20:4n-6, C20:5n-3 (EPA), C22:1, C22:6n-3 (DHA), and polyunsaturated fatty acids (PUFA) n-3, and significantly decreased (*p* < 0.05) the contents of C20:1, C20:2, C22:0, PUFA n-6/PUFA n-3 in the muscle of pigs. Our research aimed to explore the potential connections between liver status (gene expression and metabolites) and muscle fatty acids in pigs. The results of the Mantel test showed that the muscle fatty acids were closely correlated (*p* < 0.05) with liver gene expression and metabolites (Figure 6). In general, the muscle contents of C18:3n-6 and C20:5n-3 (EPA) concurrently showed significant correlations (*p* < 0.05) with liver gene expression and metabolites. In addition, the muscle contents of C20:1, C22:6n-3 (DHA), and PUFA n-3 showed significant correlations with liver metabolites (*p* < 0.05).

## 4. Discussion

Starch, as an important component of pig diet, is the main source of energy for pigs. Starches with different sources (amylose or amylopectin) had different effects on pigs, and high amylose was proved to have some beneficial effects on animal health [18]. Currently, most relevant studies focus on growth performance, meat quality, and intestinal microbiota, while studies on the liver are very few and unsystematic. Hence, in the present study, we utilized metabolomics and a transcriptome approach to estimate the impacts of diets with high-amylose-content starches on liver metabolites and gene expressions in pigs. Our results could provide a deeper understanding of the role of high-amylose-content starches in the pig industry.

### 4.1. Diets Containing Higher-Amylose-Content Starches Increased the Contents of Beneficial Unsaturated Fatty Acids and Amino Acids in the Liver of Pigs

In the present study, the results of PLS-DA and OPLS-DA suggest the liver metabolites from the two dietary treatments can be easily distinguished in the plot figures, indicating that the diets with different starch sources changed the metabolic patterns in the liver of pigs.

One of the most interesting results from our study was the increased concentration of some unsaturated fatty acids (including oleic acid, cis-9-palmitoleic acid, and α-linolenic acid) in the PS group. Oleic acid is a monounsaturated fatty acid with 18 carbon atoms. It was previously confirmed that oleic acid has multiple physiological effects, such as cholesterol metabolism, anti-inflammatory, and antioxidant [19,20]. Palmitoleic acid is a monounsaturated fatty acid that is particularly abundant in adipose tissue and liver, and cis-9-palmitoleic acid is a cis (16:1c9) isomer of palmitoleic acid [21]. Previous studies reported that palmitoleic acid had anti-inflammatory reactions when stimulated with TNF-α or palmitic acid [22,23]. Moreover, palmitoleic acid was also able to activate PPARα and play an important role in regulating glucose and lipid metabolism [24,25]. In addition, linoleic acid is an essential fatty acid for mammals and can be used as a precursor to synthesize longer-chain n-3 polyunsaturated fatty acids, docosahexaenoic acid (DHA), and eicosapentaenoic acid (EPA) [26]. We know that DHA and EPA have a wide range of physiological effects such as assisting brain cell development, anti-aging, promoting lipid degradation, and improving the health of the blood circulation system [26,27]. In addition to the above-mentioned changes in unsaturated fatty acids, we also revealed the PS diets significantly elevated the level of taurocholic acid in the liver of pigs. Taurocholic acid is one of the primary bile acids synthesized in animal liver. It was reported that taurocholic acid was not only facilitated the digestion, absorption, and excretion of lipids, but was also involved in the suppression of inflammatory cytokines [28]. The findings of earlier studies demonstrated that the PS diet or resistant starch resulted in the increased concentration of taurocholic acid in the intestine in pigs [29,30]. Hence, the increased taurocholic acid in liver after the PS diets may contribute to the lipid metabolism and health status in the present study. Overall, our results confirmed what was reported in previous studies, namely that resistant starch or starch with a high amylose content could regulate lipid metabolism and promote host health in pigs [11,31].

Our metabolomic data also indicated that the PS group elevated the concentration of valine, γ-aminobutyric acid, and carnosine in the liver of pigs. Valine, one of branched-chain amino acids (BCAAs), is the fifth limiting amino acid in a maize–soybean meal diet for pigs [32]. Meanwhile, valine plays an important role in the growth, host metabolism homeostasis, and immunity functions of human and animals [33]. γ-aminobutyric acid is an active amino acid with antioxidant and anti-inflammatory effects, and serves as a protective agent of the liver against some stimulated damages [34]. Carnosine (also called b-alanyl-L-histidine), a dipeptide with multiple biological functions, can scavenge reactive oxygen species, reactive nitrogen species, and some by-products of lipid peroxidation, and protect the host’s health by exerting its antioxidant and anti-inflammatory effects [35]. Hence, the above results suggest that PS could significantly increase some physiologically active amino acids or peptides in the liver and hence maintain the liver health of pigs.

Moreover, our results also showed that the pathways related to phosphatidylcholine biosynthesis and methylhistidine metabolism were significantly enriched, and the compounds involved in the above two pathways were phosphocholine and S-adenosylhomocysteine. The liver is an important site for phosphatidylcholine and lipoproteins synthesis. Reduced hepatic phosphatidylcholine can impair the secretion of very-low-density lipoprotein, and hence affects lipid metabolism [36]. Phosphocholine is a precursor for phosphatidylcholine synthesis [37]. Therefore, the increased phosphocholine level may promote phosphatidylcholine synthesis and contribute to lipid metabolism. Moreover, the methylation process of phosphatidylethanolamine to form phosphatidylcholine is S-adenosylmethionine-dependent [38], and S-adenosylmethionine is a universal methyl donor and can be converted into S-adenosylhomocysteine during methionine metabolism [39]. Hence, the increased S-adenosylhomocysteine indicated an increase in methyl donors in the liver of pigs after the PS diets, which indirectly contributed to the synthesis of phosphatidylcholine. However, the mechanism by which the PS diets increased methyl donors in the liver of pigs still requires further study.

### 4.2. Diets Containing Higher-Amylose-Content Starches Promoted the β-Oxidation of Fatty Acids and the Accumulation of Unsaturated Fatty Acids

Different from metabolome, transcriptome can study hose gene expressions at the mRNA level. Therefore, we conducted WGCNA on the transcriptome data to identify modules of co-expressed gene modules associated with host phenotypic traits (dietary treatments, and metabolites). Not surprisingly, our results showed that the module M11 was positively correlated with dietary treatments and 11 differential liver metabolites. Next, we outlined the main functions of M11 genes, including fatty acid degradation, tryptophan metabolism, biosynthesis of cofactors, etc. The degradation of fatty acids in the liver is closely related to its health. If the energy consumed far exceeds the amount required, the excess energy will be stored in the form of fat in adipose tissue and the liver, leading to obesity of fatty liver [40,41]. Diets containing higher-amylose-content starches have been proven to be able to regulate lipogenesis in mammalian livers [42]. Correspondingly, our results showed the liver DEGs in the M11 module identified between the two dietary treatments were mainly enriched in fatty acid degradation. Therefore, based on the above results, we believe that the diets containing higher-amylose-content starches could regulate fatty acid degradation.

We further analyzed DEGs correlated with fatty acid degradation in the present study. Our data showed the PS group elevated the expression of ACADVL, CPT1A, and MLYCD in the liver of pigs. During the lipolysis process, fatty acids must first be activated to generate fatty acyl-CoA, which then enters the mitochondria for β-oxidation. This process is an important step in lipolysis, and CPT1 is the rate-limiting enzyme for this process. CPT1 is a mitochondrial protein that catalyzes the formation of acylcarnitine from acyl groups and carnitine [43]. Consistent with our results, a previous study reported that resistant starch could elevate the expression of CPT1A in liver of rats fed high-fat diets [44]. Besides the role of CPT1, the dehydrogenation process conducted by acyl-CoA dehydrogenase may also be one of the rate-limiting steps during lipolysis [45]. ACADVL, a member of the acyl-CoA dehydrogenase family, is responsible for the initial steps in the β-oxidation of very-long-chain fatty acids, catalyzing the acyl-CoAs of 12–20 carbon in chain length in liver [46]. In addition, MLYCD can catalyze malonyl-CoA to form acetyl-CoA, thereby alleviating the inhibition of CPT by malonyl-CoA and stimulating mitochondrial β-oxidation of fatty acids [47]. Hence, the increased expressions of CPT1A, ACADVL, and MLYCD indicate the PS diets promoted the β-oxidation of long-chain fatty acids and reduced lipid accumulation in the liver of pigs.

Moreover, our results also showed that the PS diets significantly decreased the expression of CYP2U1 and ALDH1B1 in the liver when compared with the TS diets. Cytochrome P450 enzymes (CYPs) are a class of enzymes that play important roles in the synthesis and metabolism of steroids, fatty acids, and eicosanoids [48]. CYPU21 is one of the CYP isoforms, and reported to hydroxylate α-linolenic acid, arachidonic acid, EPA, and DHA, etc. [49]. In addition, ALDH1B1 is one of aldehyde dehydrogenases located in mitochondria and highly expressed in the liver of mammals, which affects liver health by regulating acetaldehyde and lipid metabolism [50]. In fasting pigs fed the same fatty acid profile diet, the above results indicated that the de novo synthesis process of fatty acids was not affected by the TS and PS diets in the present study. Combining the effects of CYP2U1 and ALDH1B1, the decrease in the expression of these two genes after being fed the PS diet may lead to an increase in the content of unsaturated fatty acids in the liver. Correspondingly, this result was consistent with our data on liver metabolites, which suggested that the content of some unsaturated fatty acids (including oleic acid, cis-9-palmitoleic acid, and α-linolenic acid) increased after the PS diets in the present study.

### 4.3. Muscle Fatty Acids Were Significantly Closely Correlated with Liver Gene Expressions and Metabolites

Liver is the main site for polyunsaturated fatty acid synthesis, de novo cholesterol synthesis, and fatty acid oxidation in pigs [51]. Therefore, the liver plays an important role in the fat deposition process in pigs. Correspondingly, our results showed that the PS diets resulted in muscles rich in unsaturated fatty acids, and the Mantel test showed muscle fatty acids were closely correlated with liver gene expression and metabolites. Our results indirectly demonstrated that the liver regulated fat deposition status in muscles of pigs, especially the unsaturated fatty acids. Numerous pieces of evidence suggest that these unsaturated fatty acids, such as DHAs and EPAs, exhibit many positive effects (such as anti-inflammation, proliferation, and differentiation) on human health [52]. Changing the fatty acid compositions of meat through the manipulation of dietary treatments has been a popular research topic in recent years. Our results confirmed that PS diets could increase the accumulation of unsaturated fatty acids in the muscle, which in turn could generate some beneficial effects on pigs and humans. It is worth noting that, although our results showed that the mechanism of causing muscle to be rich in unsaturated fatty acids may be related to metabolism and gene expressions in the liver, more systematic studies are needed to confirm this.

## 5. Conclusions

Our study utilized an integrated metabolome and transcriptome approach to evaluate the effects of diets with different starch sources on liver metabolites and gene expressions. Overall, in fasting pigs fed the same fatty acid profiles diet, the PS diets could elevate the levels of unsaturated fatty acids (such as oleic acid, cis-9-palmitoleic acid, and α-linolenic acid) and several amino acids or peptides (valine, γ-aminobutyric acid, and carnosine) in the liver. Correspondingly, the expression of fatty acid β-oxidation-related genes (such as ACADVL, CPT1A, and MLYCD) was increased, and the expression of unsaturated fatty acids metabolism-related genes (such as CYP2U1 and ALDH1B1) was decreased after the PS diets in the liver of pigs. Moreover, the above changes in the liver metabolites and gene expressions contributed to the composition of muscle fatty acids in pigs. These results provide more abundant and in-depth information on the impact of diets with higher-amylose starches on liver lipid metabolism, and offer a theoretical basis for regulating meat quality through nutritional regulation in pigs. Future systematic studies on the effects of different starch sources on lipid metabolism (diets; host metabolism; tissue deposition) in pigs are needed.

## Figures and Tables

**Table 1:** Feed ingredient and nutrient composition of experimental diets.

	Diet ^1^	
Items	TS	PS
Ingredient, %		
Tapioca starch	59	
Pea starch		59
Soybean meal	27	27
Corn gluten meal	3.4	3.4
Wheat bran	3.36	3.36
Soybean oil	3.25	3.25
L-Lysine-HCl (98%)	0.3	0.3
DL-Methionine	0.13	0.13
L-Threonine	0.06	0.06
Dicalcium phosphate	1.5	1.5
Limestone	0.3	0.3
Choline chloride (50 %)	0.4	0.4
Salt	0.3	0.3
Vitamin and mineral Premix ^2^	1	1
Total	100	100
Calculated content ^3^		
ME ^4^, MJ/kg	13.81	13.81
Standardized ileal digestible amino acids ^3^, %		
Lysine	0.88	0.88
Methionine + Cysteine	0.49	0.49
Threonine	0.5	0.5
Tryptophan	0.15	0.15
Analyzed nutrient composition		
^5^ Dry matter, %	88.48	88.45
^5^ Crude protein, %	14.54	14.55
^5^ Crude fat, %	1.26	1.25
^5^ Crude ash, %	4.09	4.11
Total starch, % DM	52.25	52.25
Amylose/amylopectin	0.11	0.44

^1^ TS: tapioca starch; CS: corn starch; PS: pea starch. ^2^ Provided per kilogram of complete diet: vitamin A, 15,000 IU; vitamin D3, 3000 IU; vitamin E, 150 mg; vitamin K3, 3 mg; vitamin B1, 3 mg; vitamin B2, 6 mg; vitamin B6, 5 mg; vitamin B12, 0.03 mg; niacin, 45 mg; vitamin C, 250 mg; calcium pantothenate, 9 mg; folic acid, 1 mg; biotin, 0.3 mg; choline chloride, 500 mg; Fe (FeSO_4_·H_2_O), 170 mg; Cu (CuSO_4_.5H_2_O), 150 mg; I (KI), 0.90 mg; Se (Na_2_SeO_3_),0.2 mg; Zn (ZnSO_4_·H_2_O), 150 mg; Mg (MgO), 68 mg; Mn (MnSO_4_·H_2_O), 80 mg; Co (CoCl_2_), 0.3 mg. ^3^ Values were estimated based on database of NRC (2012). ^4^ ME = metabolized energy. ^5^ Analytical results obtained according to AOAC (2007).

## Data Availability

The data presented in this study are available on request from the corresponding author(s).

## Supplementary Materials

The following supporting information can be downloaded at: https://www.mdpi.com/article/10.3390/ani14223192/s1, Table S1: The primers for all determined genes in the present study.
