# Supplementary material for: An Integrated Profiling of Liver Metabolome and Transcriptome of Pigs Fed Diets with Different Starch Sources
Source: Animals (Basel). 2024 Nov 7;14(22):3192. doi: 10.3390/ani14223192 (PMC11591517; doi:10.3390/ani14223192)
Supplement: Supplementary file 1 [file animals-14-03192-s001.zip › animals-3249261-supplementary.pdf]

Table S1 The primers for all determined genes in the present study.

Gene Name	Accession number	Primer sequence	Amplicon Size, bp
CYP2U1	XM_013978968.2	For: GTGGTGGTGCCGCTGTCTATTC<br>R: GCATCAGGCTCACGAACATCAGG	115
ALDH1B1	XM_003353586.5	For: ACATGAGGATCGCCAAGGAGGAG<br>R: GCAGCCGCCAAGCCATACC	115
ACADVL	XM_021067809.1	For: CTGGAGCGAGTGGAGGAGACC<br>R: GCGGGCGTACTGAGTGTTGC	114
CPTA1	NM_001129805.1	For: AGACACCATCCAGCACCTCCAG<br>R: CAACAGCCTGCCGTCGTAATAGAG	97
MLYCD	XM_021093741.1	For: CGCAGCCTCTTCCATCACATCAG<br>R: CTCTTCAGCACCCCGTTCATTTCC	140
β-actin	DQ 845171	For: CACGCCATCCTGCGTCTGGA<br>R: AGCACCGTGTTGGCGTAGAG	100
